# Distribution Patterns of Three Molecularly Defined Classes of GABAergic Neurons Across Columnar Compartments in Mouse Barrel Cortex

**DOI:** 10.3389/fnana.2019.00045

**Published:** 2019-04-30

**Authors:** Zsuzsanna Almási, Csaba Dávid, Mirko Witte, Jochen F. Staiger

**Affiliations:** ^1^Department of Anatomy, Histology and Embryology, Semmelweis University, Budapest, Hungary; ^2^Center Anatomy, Institute for Neuroanatomy, Georg-August-University Göttingen, Göttingen, Germany

**Keywords:** barrel cortex, GABAergic neuron, parvalbumin, somatostatin, vasoactive intestinal peptide (VIP), cortical laminae, septa

## Abstract

The mouse somatosensory cortex is an excellent model to study the structural basis of cortical information processing, since it possesses anatomically recognizable domains that receive different thalamic inputs, which indicates spatial segregation of different processing tasks. In this work we examined three genetically labeled, non-overlapping subpopulations of GABAergic neurons: parvalbumin- (PV+), somatostatin- (SST+), and vasoactive intestinal polypeptide-expressing (VIP+) cells. Each of these subpopulations displayed a unique cellular distribution pattern across layers. In terms of columnar localization, the distribution of these three populations was not quantitatively different between barrel-related versus septal compartments in most layers. However, in layer IV (LIV), SST+, and VIP+, but not PV+ neurons preferred the septal compartment over barrels. The examined cell types showed a tendency toward differential distribution in supragranular and infragranular barrel-related versus septal compartments, too. Our data suggests that the location of GABAergic neuron cell bodies correlates with the spatial pattern of cortical domains receiving different kinds of thalamic input. Thus, at least in LIV, lemniscal inputs present a close spatial relation preferentially to PV+ cells whereas paralemniscal inputs target compartments in which more SST+ and VIP+ cells are localized. Our findings suggest pathway-specific roles for neocortical GABAergic neurons.

## Introduction

The neocortex contains two main groups of neurons: the excitatory glutamatergic cells and the inhibitory GABAergic cells, which both are crucial for sensory information processing (Harris and Mrsic-Flogel, 2013). These two groups are molecularly, morphologically and physiologically distinct. Excitatory pyramidal cells make up approximately 80–90% of all neocortical cells and can be grouped according to laminar location and projection targets (Huang, 2014; Harris and Shepherd, 2015). Inhibitory GABAergic neurons comprise only about 10–20% of the total population of neocortical neurons, but their diversity is surprisingly large (Markram et al., 2004; Rudy et al., 2011; De Felipe et al., 2013; Tremblay et al., 2016; Feldmeyer et al., 2018). The parvalbumin (PV+), somatostatin (SST+), and vasoactive intestinal polypeptide (VIP+) expressing cells account for the majority of GABAergic neurons (roughly 40% PV, 30% SST, and 10–15% VIP). These three distinct classes show minimal overlap in all examined cortical areas (Pfeffer et al., 2013; Tremblay et al., 2016), and have diverse molecular, structural and electrophysiological features. However, their detailed distribution and specific functions are largely unknown. A non-uniform distribution would imply distinct functions in local circuits of the barrel cortex (Feldmeyer et al., 2013). In this frame, we described the spatial distribution of these neuron types, in order to investigate their relationship to cortical compartments receiving different thalamic afferentation.

Sensory information from the whisker pad is transmitted via modularly organized parallel pathways to the barrel cortex (Diamond et al., 2008; Feldmeyer et al., 2013; Zembrzycki et al., 2013). The trigeminal nerve projects to several nuclei in the brainstem, namely the mesencephalic, principal (or pontine) and spinal nucleus. The lemniscal pathway originates in the principal trigeminal nucleus and relays tactile information via the barreloids of the ventral posteromedial nucleus (VPM) of the thalamus. The paralemniscal pathway routes its information through the spinal trigeminal nucleus (intermediate part) and medial part of the posterior thalamic nucleus (POm). From thalamus, ascending information reaches different cortical areas and layers. The primary site of termination is the barrel cortex, where the lemniscal thalamocortical fibers strongly project to layer IV (LIV) and thus also define supragranular and infragranular compartments, which are radially aligned with a barrel (Woolsey and van der Loos, 1970). This leads to the formation of barrel-related columns that are driven by their corresponding whiskers (Staiger et al., 2002; Wagener et al., 2016). Barrel columns are separated by septal compartments (Kim and Ebner, 1999; Alloway et al., 2004). The lemniscal pathway projects via VPM to the barrel columns, most strongly into LIV, layer Vb (LVb), layer VI (LVI), and less strongly to layer II/III (LII/III) (Lu and Lin, 1993; Bureau et al., 2006; Oberlaender et al., 2012), whereas the paralemniscal pathway projects via POm preferentially to layer I (LI) and layer Va (LVa) in a column-overarching manner and sends a few fibers to the LIV septum (Alloway, 2008; Wimmer et al., 2010b).

Single-cell recordings *in vivo* and *in vitro*, combined with biocytin filling and morphological reconstruction, revealed functional and morphological features of the different GABAergic neurons, found in the three subpopulations (Markram et al., 2004; Jiang et al., 2015; Tremblay et al., 2016; Feldmeyer et al., 2018). PV+ cells are located in all cortical layers (but LI). They prefer layers IV and Vb, however (Celio, 1986). Their dendritic and axonal arbors can show diverse patterns, depending on the precise laminar location of the soma; in this home layer the densest axonal as well as dendritic arborization can be found (Wang et al., 2002; Munoz et al., 2014; Koelbl et al., 2015). Their inhibitory influence strongly affects their own population (Tamas et al., 2000; Staiger et al., 2009; Pfeffer et al., 2013), but they can effectively inhibit other GABAergic interneuron types (David et al., 2007; Jiang et al., 2015; Karnani et al., 2016b; Walker et al., 2016). Furthermore, they are classically considered to be (together with the rare axo-axonic cell) the most effective inhibitors of pyramidal cells (Kubota et al., 2015; Neske et al., 2015).

SST+ cells show a bias toward infragranular layers. However, the different subpopulations, namely Martinotti and non-Martinotti cells, can show very distinct patterns of soma localization and axonal targeting (Oliva et al., 2000; Ma et al., 2006; Urban-Ciecko and Barth, 2016; Nigro et al., 2018). Compared to PV+ cells, they rather seem to avoid inhibiting other SST+ cells, but impose strong inhibition on other interneurons and pyramidal cells, in a cell type-specific manner (Caputi et al., 2009; Pfeffer et al., 2013; Xu et al., 2013). To exert their likely distal dendritic inhibition, they send dense axonal projections strongly but not exclusively into LI whereas their dendritic arborization is far less wide-ranging but usually not restricted to the home layer (Ma et al., 2006; McGarry et al., 2010; Nigro et al., 2018).

By contrast, VIP+ cells show a preferential location in the supragranular layers of mouse and rat barrel cortex (Bayraktar et al., 2000; Prönneke et al., 2015) and have recently been implicated in parallel disinhibitory and inhibitory circuits, impinging on pyramidal cells (Lee et al., 2013; Pi et al., 2013; Garcia-Junco-Clemente et al., 2017; Kuchibhotla et al., 2017; Zhou et al., 2017). In terms of input-output patterns, we have recently suggested that L II/III VIP+ cells have a dendritic tree that is largely restricted to L I-III but an axonal arbor that reaches all layers of a barrel-related column. On the other hand, VIP+ neurons in L IV-VI display a variable dendritic tree that could span all layers, whereas the axon is basically confined to infragranular layers (Prönneke et al., 2015). A major connectivity motif is VIP+ cells inhibiting SST+ Martinotti cells, in barrel as well as visual cortex (Caputi et al., 2009; Pfeffer et al., 2013; Karnani et al., 2016a; Walker et al., 2016).

As already noted above, thalamocortical projections and interneuron somata show layer preferences. However, there are also columns as another organizational principle of the cortex (Mountcastle, 1997). Although a barrel-related column preference of lemniscal and a septal compartment (often also called column) preference of paralemniscal thalamocortical fibers are well described, there is very little information available about the barrel- or septum-related columnar preferences of GABAergic neurons. There are only few cases, where not only the laminar, but the horizontal distribution was examined (Hajós et al., 1998; Nogueira-Campos et al., 2012). It is known that different types of interneurons can be the target of thalamocortical fibers of different origin (Staiger et al., 1996a,b; Porter et al., 2001; Cruikshank et al., 2010; Ji et al., 2016; Audette et al., 2017; Maffei, 2017). However, a more refined knowledge on the distribution pattern could help design experiments to further analyze thalamic inputs to GABAergic neurons in more detail.

Thus, in the present study, we aimed to answer the question whether PV+, SST+, and VIP+ neurons show a preference for the laminar and columnar compartments of the barrel cortex, as defined by the two main thalamocortical pathways. Since LIV has a unique and central role as an access point of tactile information to the cortex and the barrel versus septum distinction is most obvious there, we put a special focus on this layer. The main question thus was, whether a compartment-associated distribution of GABAergic neurons can be found? Our results show that although laminar and columnar preferences obviously do exist for the soma locations of PV+, SST+, and VIP+ cells, only in LIV and only for SST+ and VIP+ cells, these differences reach statistical significance.

## Materials and Methods

### Animals

Three different genetically engineered mouse strains were used for the present experiments: (1) a cross breed of PVcre/tdTomato mice (crossed B6;129P2-Pvalbtm1(cre)Arbr/J with B6.Cg-Gt(ROSA)26Sortm9(CAG-tdTomato)Hze/J mice) and GIN (Oliva et al., 2000) mice (PV-GIN; *n* = 6, male), which expressed the red fluorescent protein tdTomato in PV cells and green fluorescent protein (GFP) in a subset of SST cells, (2) SSTcre/tdTomato mice (crossed Ssttm2.1(cre)Zjh/J with B6.Cg-Gt(ROSA)26Sortm9(CAG-tdTomato)Hze/J mice) (*n* = 7, male) were used for visualizing all somatostatin cells, and (3) VIPcre/tdTomato mice (crossed VIPtm1(cre)Zjh with B6.Cg-Gt(ROSA)26Sortm9(CAG-tdTomato)Hze/J mice) (*n* = 9, male), were used to label all vasoactive intestinal polypeptide (VIP) cells. The age of animals was between 6 and 8 weeks. All used animals were obtained from the breeding facility of the University Medical Center Göttingen (Germany). Animal numbers and their suffering were restricted to the minimum. This study was carried out in accordance with the principles of the Basel Declaration and German laws on animal research (TierSchG and TierSchVersV, 2013). The protocol was approved by the LAVES (Niedersächsisches Landesamt für Verbraucherschutz und Lebensmittelsicherheit).

### Tissue Preparation and Immunocytochemistry

The animals were anesthetized with an overdose of ketamin (Essex Tierarznei) and transcardially perfused with 0.9% NaCl solution to remove blood from vessels, followed by 4% paraformaldehyde dissolved in 0.1 M phosphate buffer (PB, pH 7.4). The brains were removed and the two hemispheres separated. The left hemispheres were flattened (Welker and Woolsey, 1974) whereas the right ones were kept unmodified, and all tissue was postfixed for 2 h in the same fixative. The right hemispheres were cut in the coronal plane, the left hemispheres tangentially on a vibratome (VT 1200S, Leica), both with a nominal section thickness of 50 μm.

The sections of PV-GIN animals were incubated with rabbit anti-GFP (Invitrogen) 1:2500 in TRIS-buffered saline with 0.3% Triton X-100 (TBST) for 2 days (in a cold room), followed by an anti-rabbit IgG coupled with Alexa-488 (Invitrogen) 1:500 in TBST for 2 h. The PV+ cells contained a sufficient amount of tdTomato, therefore they did not need further signal amplification. The sections of SSTcre/tdTomato and VIPcre/tdTomato animals were stained with guinea pig anti-vesicular glutamate transporter 2 (vGlut2; Millipore) 1:10,000 in TBST for 2 days, followed by an anti-guinea pig IgG coupled with Alexa 488 (Invitrogen) 1:500 in TBST for 2 h, in order to identify barrels. In a last step, sections of all strains were stained with DAPI (1:1000, Molecular Probes) to label cell nuclei. The sections were then embedded in AquaPolymount (Polysciences).

### Image Acquisition

Microphotographs were taken with an epifluorescence microscope (AxioImager.M2, Zeiss, Jena, Germany; 5x A-Plan objective, NA 0.12 or 10x C-Plan Neofluar objective, NA 0.3) as “virtual tissue photomontages” controlled by Neurolucida software (MBF Bioscience, Colchester, VT, United States). Filter sets used were: for DAPI #49 (BP 365; BS 395, BP 445/50), for Alexa488/GFP #44 (BP 475/40; BS 500, BP 530/50), and for tdTomato #45 (Bp 560/40; BS 585, BP 630/75. To maintain illumination intensity comparable across sections, the dynamic range was set according to the saturation of a few (3–5) brightest pixels that belonged to somata.

### Data Extraction

Please consult Supplementary Figure 1 for a more intuitive illustration of the work flow. Consecutive tangential sections were aligned according to the pattern of vertically oriented cortical blood vessels. Neurolucida was used for registering cells and cortical domains. First, the barrels were identified and labeled individually, based on DAPI staining that was correlated with either intense PV neuropil or vGluT2 immunostaining, which is caused by thalamocortical projections into layer (L) IV. In coronal sections, the borders between LI, LII/LIII and LVa, LVb, and LVI were identified on the basis of DAPI staining. The pia mater and the LVI/white matter border were also delineated throughout the entire barrel field. Then, the location of all PV, SST, and VIP cells was registered independent of the laminar boundaries, thus the borders of the cortical domains did not bias the data collection. The resulting data files were analyzed in Neurolucida Explorer (MBF Biosciences). The following parameters were extracted: (i) cell numbers, (ii) areas of all delineated cortical domains (barrels, septa, and the related layers of the respective cortical columns), and (iii) the coordinates of all the GABAergic neurons with their distance from pia mater and white matter.

### Data Processing

After identifying each barrel in coronal sections, the depth of the borders between cortical layers was measured for each, and the medio-lateral and rostro-caudal coordinates were also registered. These parameters were used to correct the measured volumina of the flattened tangential sections. After that procedure, the tangential sections were re-binned into 20 sections. In coronal sections we found the cortical thickness to be 1019,164 ± 38,550 μm. In case of 50 μm-thick sections it would contain a total number of ca. 20 tangential sections. By cutting the brain tangentially, we had diverse section numbers (15–19), depending on applied pressure during flattening. Therefore, we standardized the cortical thickness through re-binning the sections with the following methods. First we divided each section in 20 virtual subsections and divided the cell count of a given section equally between its virtual subsections. For example, in an animal with *n* = 17 sections (*n* = original section number), we got *n*’ = 340 subsections (*n*’ = number of a new subsection, *n*’ = *n*^∗^ 20). Then, we created normalized tangential sections: one 20th of n’ of all subsections were binned together (in the example above, 17 subsections were binned in one new normalized section (Supplementary Figure 2).

The densities of cells of interest (PV+, SST+, VIP+) were plotted and the borders of cortical layers were defined, using the inflection points of these curves (Pohlkamp et al., 2014). Since the cortical and laminar thickness varies according to rostro-caudal and medio-lateral position, the extracted raw data had to be transformed to obtain statistically comparable quantities. The normalization of cortical thickness utilized landmarks, e.g., points of inflection on laminar borders as described previously for each of the markers examined (Pohlkamp et al., 2014). This normalization process transformed all the data into the same thickness range, therefore it made the comparison (or pooling) of coronally- versus tangentially-cut as well as more rostrally or medially versus more caudally or laterally located tissue possible.

To determine whether our data showed Gaussian distribution, we used the Shapiro–Wilk normality test. Since the Shapiro–Wilk test failed in all cases of studied cell types, the non-parametric Kruskal–Wallis test was used to determine statistically significant differences. Alpha levels were adjusted with the Bonferroni method. For comparison of numbers from tangential versus coronal sections, data were plotted and the correlation coefficient was calculated (Mystat, Systat Software, Inc., United States). We collected data from coronal sections to compare our results to previous literature. Tangential section were used to collect data about absolute numbers in the whole posteromedial barrelfield and because the identification of barrels and interbarrel septa was much more obvious. Coronal and tangential data series correlated highly with each other, therefore the density values of coronal and tangential section were pooled for the analysis.

## Results

### PVcre/tdTomato-Expressing (PV+) GABAergic Neurons

**Figure 1:**
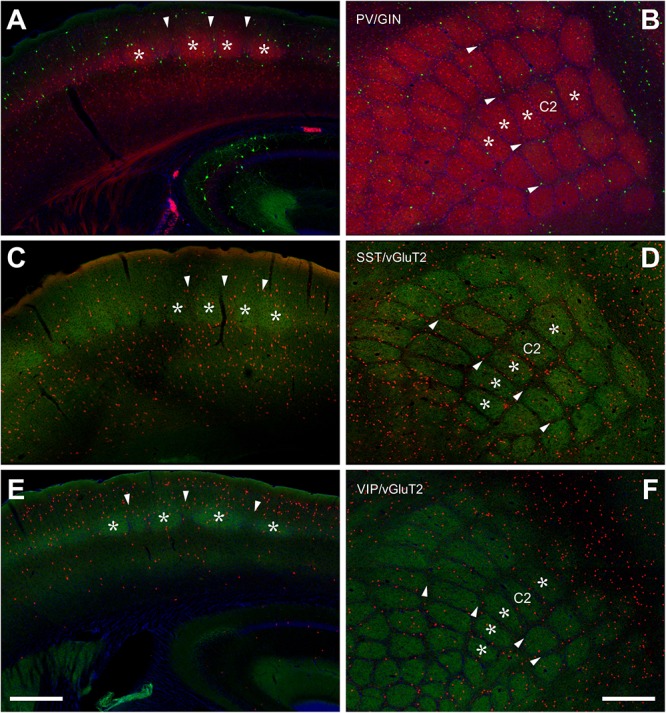
Localization of PV+, SST+, and VIP+ interneurons in the barrel cortex. **(A,B)** Double labeling of PV+ (red, from PVcre/tdTomato animals) and SST+ (green, GIN line) shows a clear delineation of barrel-related columns in the coronal section **(A)** and a whisker-related somatotopic pattern in layer IV of tangential sections **(B)**. Indication of barrels (*asterisks*) and septa (*arrowheads*) holds for all images. PV+ cells seem to prefer barrels, but quantitative analysis showed that the cell bodies are distributed homogenously. GIN cells, however, displayed an obvious preference for the septal compartment. **(C,D)** SST+ cells (red, from SSTcre/tdTomato animals) were located preferentially in the infragranular layers **(D)**, somewhat aligned to interbarrel septa **(C,D)**. VGluT2 (green) strongly labels the lemniscal thalamic termination zone in layer IV barrels. **(E,F)** VIP+ cells (red; from VIPcre/tdTomato animals) preferred supragranular layers **(E)** and interbarrel septa **(F)**. Scale bars: 500 μm.

These cells show a dominant location within the termination zones of the lemniscal thalamic projections (Chmielowska et al., 1989; Wimmer et al., 2010b), namely LIV and the LVb/VI border but also within the termination zone of paralemniscal thalamic projections (Wimmer et al., 2010b), namely layer Va (Figure 1A). Indeed, 26.17 ± 2.73% of all PV+ cells were located in LIV, 14.84 ± 1.94% in LVa and 27.15 ± 1.49% in LVb, whereas in layers II/III and VI together only 31.83 ± 1.99% were found. In LIV, the neuropil labeling led to the clear delineation of barrels, in coronal as well as tangential sections (Figure 1A,B).

### Laminar Distribution

We studied the PV+ cell distribution in coronal sections of 3 hemispheres and in tangential sections of another 3 hemispheres. The coronal and tangential sections correlated with each other in terms of cell number and distribution (*n* = 6; *r*^2^ = 0.925) (Figure 3D), therefore they were pooled for further analysis (Table 1 and Figure 3A).

**Table 1:** Cell density of PV-expressing neurons, differentiated by barrel column and septum.

	Cell density in column (1/mm^3^)	± SD (1/mm^3^)	Cell density in septum (1/mm^3^)	± SD (1/mm^3^)
Layer I	0	0	0	0
Layer II/III	4995.65	1278.58	5050.46	2012.55
Layer IV	8426.59	1405.35	8252.19	1751.12
Layer Va	8510.96	1766.67	7904.56	2217.68
Layer Vb	8964.038	2143.11	8244.73	3170.58
Layer VI	3017.66	1003.13	2766.40	877.38

When extrapolated in number to 1 mm^3^, PV+ cells preferred LIV and LV (a and b) in approximately equal numbers, were much sparser in LII/III and LVI and avoided LI (Table 1 and Figure 1A,B, 3; significance levels are listed in Supplementary Table 1, Kruskal–Wallis test was used to determine statistically significant differences). Thus, PV+ cell numbers showed a differential distribution between layers, which was very similar in the two analyzed columnar compartments (see also below).

### Columnar (Barrel Versus Septum) Distribution

**Figure 2:**
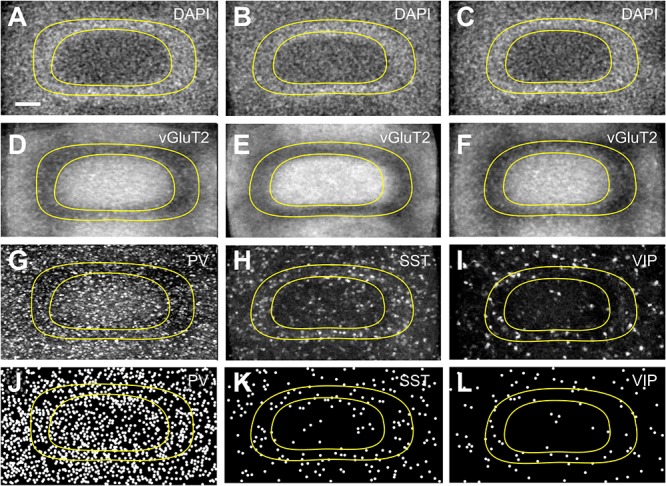
Distribution of interneurons across layer IV barrels and septa. Fine-grained distribution of **(A,D,G,J)** PV+, **(B,E,H,K)** SST+, and **(C,F,I,L)** VIP+ interneuron somata, using tangential sections to allow a better delineation of septal and barrel compartments. DAPI staining **(A–C)** or vGluT2 immunostaining **(D–F)** was used to reveal the border of the barrels in a complementary manner. Images of nominally 50 μm thick sections of PV+ **(G)**, SST+ **(H),** and VIP+ **(I)** somata and neuropil, in conjunction with the respective Neurolucida reconstructions **(J–L)** reveal a homogenous distribution of PV+ as well as a septal preference of SST+ and VIP+ cells. Since SST and VIP neurons appear in a quite low density, especially in the granular layer, we had to project numerous barrels onto each other, in order to be able to display the uneven distribution of these cells. For that purpose, we collected barrels, which could be fit into an idealized (averaged) barrel, and then the images were projected to a single image plane using ImageJ Sum Slices function. This was not necessary for PV cells since the cell density was much higher, but for the sake of comparability we performed the same protocol on PV images. Scale bars: 50 μm.

**Figure 3:**
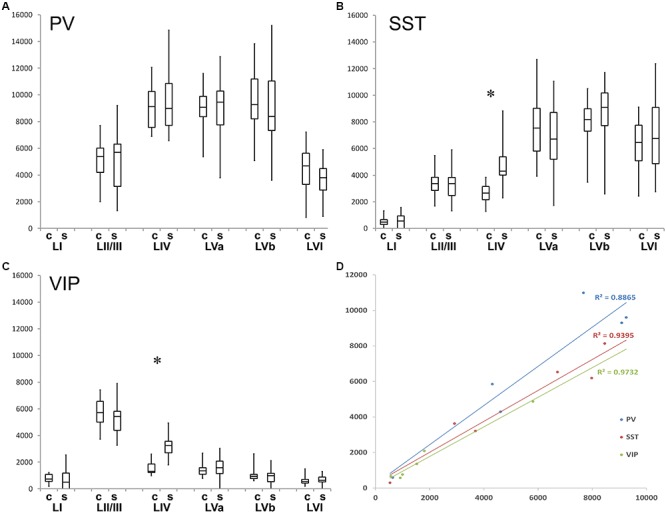
Quantification of layer- and column-related distribution of GABAergic interneurons. Box and whisker plot of laminar density of **(A)** PV+, **(B)** SST+, and **(C)** VIP interneurons. Note the statistically significant difference between barrel column (C)- and septum (S)-associated compartments for VIP+ and SST+ cells in layer IV. Significant laminar distribution differences were not denoted because of figure transparency, details were explained above. The graphs also show that all of the studied interneurons completely (PV+) or largely (SST+, VIP+) avoid LI. **(D)** Diagram shows the cell densities of coronal versus tangential sections. Correlation coefficients (R) are close to 1, so the numbers are highly correlated to each other (R_PV_. = 0.925; R _SST_ = 0.940; R _V IP_ = 0.973).

In layers II throughout VI, the distribution of PV+ cells was found to be similar for barrel- and septum-related compartments. However, this more or less even distribution of cell bodies was masked by the differential distribution of PV+ neuropil, which strongly preferred barrels (Figure 1B and Table 1). There were slightly more cells in barrels versus septa (Figure 2A,D,G,J, 3A, LIVc versus LIVs), but this difference was not statistically significant (*p* = 0.885; significance levels are listed in Supplementary Table 2, Kruskal–Wallis test was used to determine statistically significant differences). Considering soma and neuropil labeling in PV+ neurons, in LIV this resulted in a complementary distribution profile, contrasting with VIP+ and SST+ GABAergic neurons (see below).

### SST-Expressing (SST+) GABAergic Neurons

The overall distribution of SST+ cells was unique in several aspects (Figure 1C,D). Whereas PV+ cells showed an increased density in layers IV and Vb (as noted above), SST+ cells strongly preferred infragranular layers V-VI. In fact, 73.69 ± 5.37% of the cells were located in the infragranular layers, whereas only 26.31 ± 5.37% were found in layers I-IV. This suggests that projections like the one from M1 with heavy terminal labeling in infragranular layers might be a preferential input to these neurons (Kinnischtzke et al., 2014) but also that paralemniscal inputs have ample opportunity in targeting this class of cells in LI and LVa, whereas lemniscal inputs should preferentially innervate cells in LVb/LVI (Wimmer et al., 2010b; Audette et al., 2017).

### Laminar Distribution

We studied the SST+ cell distribution in coronal sections of 3 hemispheres and in tangential sections of another three hemispheres, too. The coronal and tangential sections (total *n* = 6) correlated highly with each other (*r*^2^ = 0.94) (Figure 3D), therefore the neurons were pooled for further analysis (Table 2 and Figure 3B).

**Table 2:** Cell density of SST-expressing neurons, differentiated by barrel column and septum.

	Cell density in column (1/mm^3^)	± SD (1/mm^3^)	Cell density in septum (1/mm^3^)	± SD (1/mm^3^)
Layer I	512.67	356.23	566.29	558.33
Layer II/III	3353.63	903.46	3318.67	1159.57
Layer IV	2661.42	697.03	4772.67	1535.58
Layer Va	7594.97	2459.81	6752.20	2268.18
Layer Vb	8064.99	1587.00	8675.49	2425.20
Layer VI	6344.08	1985.93	6856.99	2454.67

The SST+ neurons were similar in number in layers Va, Vb, and VI. Each of these layers possessed significantly more cells than LIV, LII/III, and LI, respectively. LII/III SST+ cells did not differ in number from LIV cells. The lowest number of SST+ cells was found in LI, which was significantly different from all other layers (significance levels are listed in Supplementary Table 3; Kruskal–Wallis test was used to determine statistically significant differences).

### Columnar (Barrel Versus Septum) Distributions

When comparing the barrel with the septal compartment, we found that SST+ cells were distributed more or less homogenously between these compartments in all layers, except LIV (Table 2). As shown in Figure 2B,E,H,K, 3B, in LIV, the septal compartment contained significantly more cells than the barrel compartment (*p* < 0.0001) (significance levels are listed in Supplementary Table 4; Kruskal–Wallis test was used to determine statistically significant differences).

### VIP-Expressing (VIP+) GABAergic Neurons

Interestingly, VIP+ cells show an inverse relationship to SST+ cells, as they display a strong preference for the supragranular layers (Figure 1E). Indeed, 70.24% of all cells were located in layers I-III, whereas only 29.76% were found in layers IV-VI. Since the subpopulation located in LI and LII/III shows extensive dendritic arborization in layer I (Prönneke et al., 2015), projections like those from motor cortex or basal forebrain can strongly target these cells (Mechawar et al., 2000; Lee et al., 2013), as does the lemniscal thalamus in deep LII/III (Staiger et al., 1996b; Wall et al., 2016).

### Laminar Distribution

We studied the VIP+ cell distribution in coronal sections of three hemispheres and in tangential sections of another three hemispheres. The cell numbers in these sections (total *n* = 6) were again correlated highly with each other (*r*^2^ = 0.973) (Figure 3D), thus, the values were pooled for further analysis (Table 3 and Figure 3C).

**Table 3:** Cell density of VIP-expressing neurons, differentiated by barrel column and septum.

	Cell density in column (1/mm^3^)	± SD (1/mm^3^)	Cell density in septum (1/mm^3^)	±SD (1/mm^3^)
Layer I	788.87	335.88	571.67	536.68
Layer II/III	5596.69	968.70	5150.93	1090.85
Layer IV	1574.74	481.32	3204.72	908.99
Layer Va	1448.33	492.38	1594.11	709.10
Layer Vb	1007.70	454.04	852.38	596.40
Layer VI	630.11	285.53	672.18	313.03

VIP+ cells in LII/III outnumbered those in all other layers significantly whereas LI had significantly lower numbers than all other layers (except LVI). Also the gradient from LIV to LVI reached statistical significance, with each deeper layer housing less neurons than the one located pialward (significance levels are listed in Supplementary Table 5; Kruskal–Wallis test was used to determine statistically significant differences).

### Columnar (Barrel Versus Septum) Distribution

When comparing the columnar and septal compartments, there was no obvious difference in the supragranular and infragranular layers. However, in LIV we found significantly more cells in septa than in barrels (*p* < 0.0001; Kruskal–Wallis test was used to determine statistically significant differences), which can be seen in Figure 1F, 2C,F,I,L, 3C. This columnar segregation was therefore restricted to the granular layer where the cell density in septa was approximately two times higher than in barrels (significance levels are listed in Supplementary Table 6; Kruskal–Wallis test was used to determine statistically significant differences).

In summary, we could reproduce the general pattern of a dominance of PV+ cells in lemniscal thalamorecipient layers (LIV and Vb) but show a frequent occurrence on the paralemniscal thalamorecipient LVa, too. SST+ showed a strong abundance in LVa/b and LVI whereas VIP+ were preferentially distributed in LII/III and LIV (Supplementary Figure 3). In addition, when comparing barrel-related columns with septum-associated compartments, only in LIV we could detect a statistically significant preference of SST+ and VIP+ cells for the latter (Supplementary Figure 3).

## Discussion

In the present study, we confirmed and extended the laminar distribution profiles of major subpopulations of GABAergic neurons in the mouse barrel cortex (for recent reviews see Staiger et al., 2015; Tremblay et al., 2016; Feldmeyer et al., 2018). In agreement with these previous studies, we show in tdTomato-expressing mouse lines that PV+ cells prefer layers IV and Vb, whereas SST+ cells prefer all infragranular and VIP+ cells supragranular (LII/III) layers. We extend these findings by showing that PV+ cells do not have a preferential localization within columnar compartments, since cell counts show a very similar distribution between barrel column- and septum-associated compartments. Thus, it is an anisotropic distribution of PV+ neuropil labeling that accentuates barrels in coronal as well as tangential sections. On the other hand, SST+ and VIP+ cells were significantly more frequent in the septum than the barrel (in LIV), whereas this unique distribution was just a trend, if anything, outside of LIV. These findings led us to propose that different subpopulations of neocortical GABAergic neurons could be differentially associated with different input pathways, as initially suggested for the archicortex (Somogyi et al., 1998). This notion was also referred to in the sensorimotor cortex (Kubota et al., 2007; Cruikshank et al., 2010; Audette et al., 2017), although these, as well as more recent studies in auditory and visual cortex, did not reveal a very refined picture (Ji et al., 2016). Since it is obvious that soma location is only a poor predictor for the entire input space of a neuron, we and others are currently trying to substantiate the hypothesis of thalamic (and other) input specificity on different classes of GABAergic neurons with optogenetics and whole cell recordings.

### Technical Considerations

We compared and finally pooled results obtained from coronal and tangential sections of the barrel cortex (Woolsey and van der Loos, 1970). The advantage of frontal sectioning is that laminar borders can be identified with high certainty, whereas in tangential sections the barrel versus septal regions can be delineated unequivocally. Since tangential sectioning requires a certain compression of the cortex, and the cortex is not evenly thick along its medio-lateral axis, the distance of the cells from the pia mater had to be adjusted, in order to match laminar location in tangential with coronal sections. After adjustment, the cell density read-out from coronal and tangential sections was virtually identical, allowing us to pool the data. Our laminar density results of VIP+ cells are in very good agreement with that previously described for the barrel cortex (Prönneke et al., 2015) and with PV+ and SST+ cell counts from other ongoing projects (own unpublished results).

Here, we used staining for vGlut2 and DAPI as pre- and postsynaptic markers, respectively, to determine barrel and septum borders, building on our previous experience (Wagener et al., 2010). Because of putative plasticity of thalamocortical fibers (Wimmer et al., 2010a), the vGlut2-defined barrel borders could vary, thus the additional use of DAPI staining is important to define the actual barrel size and further helps to distinguish barrel and septal compartments (Erzurumlu and Kind, 2001).

It should also be noted that we have counted tdTomato-expressing cell bodies distributed across the various layers and columns, which does not necessarily mean that they are specifically and comprehensively reflecting the entire population of interest, as represented by the different cre-driver lines. However, previous studies already showed a reasonable overlap of tdTomato-fluorescence with the respective markers (GAD1, PV, SST, and VIP (Taniguchi et al., 2011; Pfeffer et al., 2013); and our own published and still ongoing efforts to characterize these mouse lines revealed a very high specificity and an excellent sensitivity (Prönneke et al., 2015).

### Comparison to Previous Studies

To the best of our knowledge, the numbers of GABAergic neurons in mouse barrel cortex had not been established before and the partitioning of GABAergic neurons between barrel- and septal column was completely lacking. It has to be acknowledged that there is a comprehensive quantification with an immunohistochemical approach available, the area-densities reported there being in general agreement with our results (Xu et al., 2010). Here, we started to overcome this lack of detailed knowledge by counting soma numbers of neurons expressing tdTomato-fluorescence under the control of three marker genes that are currently viewed as the best candidates to separate the population of GABAergic neurons into three different classes (Taniguchi et al., 2011; Tremblay et al., 2016).

In PVcre/tdTomato mice, we could detect a distribution across layers that was both, qualitatively and quantitatively similar to the rat somatosensory cortex (van Brederode et al., 1991; Ren et al., 1992). We showed numbers peaking in layers IV, Va, and Vb, which would mean that major layers receiving lemniscal or paralemniscal input (Wimmer et al., 2010b) house a feedforward inhibition motif in their circuitry (Cruikshank et al., 2010; Naka and Adesnik, 2016), which does strongly influence their *in vivo* physiology (Bruno and Sakmann, 2006; de Kock et al., 2007; Yu et al., 2016).

Surprisingly, in tangential sections we did not find a significant difference between barrel and septal compartments, although PV has been used as a marker for barrels (Sukhov et al., 2016). This means that barrels are highlighted in PV staining by virtue of the neuropil labeling (mainly being axonal boutons) and not by somatodendritic profiles. This could be explained by anisotropic axonal arbors of basket cells displaying a bias toward barrel centers (Munoz et al., 2014; Koelbl et al., 2015). Further evidence that there is a profound difference between the organization of PV+ cells’ dendrites versus axons in barrels versus septa comes from a recent study that showed their gap junction coupling to occur in a cell type- and location-specific manner (Shigematsu et al., 2018).

In SSTcre/tdTomato mice, we were able to show that all infragranular layers express high numbers of these cells, where they recently have been shown also to be most diverse in structure and function (Nigro et al., 2018). In contrast, the fewer cells located in LII/III all seem to be Martinotti cells (Munoz et al., 2017; Nigro et al., 2018), which was also consistently reported in the GIN mouse line (Oliva et al., 2000; Ma et al., 2006; Walker et al., 2016). The minority population in LI remains to be better characterized (Ma et al., 2014).

The analysis in tangential sections, revealing a preferential location in septa when compared to barrels, is without precedent. One has to know that septa in mice are very small compartments, as compared to rats, thus it is very difficult to specifically record from them, in order to assess their function (Welker and Woolsey, 1974). We can only speculate that the increased number of SST+ neurons (but also VIP+ cells, see below) might serve to flexibly adjust the receptive field size of septal neurons, which is usually larger than that of barrel-related column neurons (Brecht and Sakmann, 2002).

In VIPcre/tdTomato mice, we obtained cell numbers across the different layers that were in good agreement with previous reports (Xu et al., 2010; Prönneke et al., 2015), pinpointing a preferential role of these neurons to relay information originating from diverse sources via axonal projections into layers I-III. This information is then relayed to all layers of the respective barrel column- or septum-associated compartment by virtue of their “column-filling” axonal arbors, probably impinging on excitatory and inhibitory neurons in parallel (Garcia-Junco-Clemente et al., 2017; Kuchibhotla et al., 2017; Zhou et al., 2017).

As noted already above, a novel finding was the differential distribution of VIP+ somata in barrels versus septa, with approximately twice as many cells in the septa than in the barrels. Interestingly, a previous study has reported a differential distribution of VIP+ boutons, which were more numerous in the side region than within the hollow (Zilles et al., 1993). Since a “side” is the barrel wall and the septum pooled (Welker and Woolsey, 1974) and since VIP neurons in layer II/III and IV have a radially restricted axonal arbor (Prönneke et al., 2015), these findings are complementary to ours.

### Functional Implications

As noted above, the soma location alone is a very limited predictor of the input space of a neuron. However, since the dendritic arbors of GABAergic neurons are usually more compact than those of pyramidal cells, this could serve as a reasonable first approximation to consider the layer- and column-associated location of the soma as the main input space of the respective neuron (De Felipe et al., 2013).

Thus, we suggest that PV+ neurons are more or less uniformly distributed to fulfill the need of all cortical circuits to operate in a tight excitation-inhibition-balance (Isaacson and Scanziani, 2011), no matter whether feedforward pathways from the thalamus or local pathways activate them. SST+ cells, with their strong bias to infragranular layers and to LIV septa, are rather heterogeneous in their input and output domains, making a more specific functional interpretation than that they are providing widespread dendritic inhibition difficult (Urban-Ciecko and Barth, 2016; Yavorska and Wehr, 2016). VIP+ neurons, with their strong bias to supragranular layers and also LIV septa, can directly participate in sensory processing, potentially by strong disinhibitory mechanisms (Karnani et al., 2016a,b; Walker et al., 2016; Feldmeyer et al., 2018) but they also relay more global salience- or reward-related signals, originating in (pre-)motor cortex or in subcortical neuromodulatory centers to the column (Lee et al., 2013; Kepecs and Fishell, 2014).

The enrichment of SST+ and VIP+ neurons in LIV LIV septa might help to task-dependently switch cortical activity flow (and thus sensory processing) between the nested, partly distinct, partly overlapping, circuits that originate in or are wired through barrel- versus septum-associated compartments of the barrel cortex (Alloway, 2008; Diamond et al., 2008; Feldmeyer et al., 2013). The next task will be to perform optogenetic stimulation of these putative input pathways and record from single identified GABAergic neurons, in order to test our predictions.

## Author Contributions

ZA and CD conceived and performed the experiments, analyzed the results, and co-wrote the manuscript. MW provided the materials and co-wrote the manuscript. JS conceived the experiments, supervised the project, and wrote the manuscript.

## Conflict of Interest Statement

The authors declare that the research was conducted in the absence of any commercial or financial relationships that could be construed as a potential conflict of interest.
