# Supplementary material for: Distribution Patterns of Three Molecularly Defined Classes of GABAergic Neurons Across Columnar Compartments in Mouse Barrel Cortex
Source: Front Neuroanat. 2019 Apr 30;13:45. doi: 10.3389/fnana.2019.00045 (PMC6503091; doi:10.3389/fnana.2019.00045)
Supplement: Supplementary file 1 [file Data_Sheet_1.docx]

**Supplementary material**

|  | LI | LII/III | LIV | Lva | LVb | LVI |
| --- | --- | --- | --- | --- | --- | --- |
| LI |  | 0,0001 | 0,0001 | 0,0001 | 0,0001 |  |
| LII/III |  |  | 0,01 | 0,025 | 0,016 | 0,025 |
| LIV |  |  |  | 0,631 | 0,873 | 0,004 |
| Lva |  |  |  |  | 0,522 | 0,004 |
| LVb |  |  |  |  |  | 0,004 |
| LVI |  |  |  |  |  |  |

**Supplementary table 1:** The levels of significance (p) for a comparison of layers. Alpha was calculated by Bonferroni adjustment. Alpha= 0,0033^.^ in case of PV+ cells. Kruskal-Wallis test was used to determine statistically significant differences.

|  | Column vs. Septum |
| --- | --- |
| LI | 0,228 |
| LII/III | 0,513 |
| LIV | 0,885 |
| Lva | 0,678 |
| LVb | 0,672 |
| LVI | 0,080 |

**Supplementary table2:** This table shows the levels of significance (p) for a comparison of septal vs barrel compartments. Alpha was calculated by Bonferroni adjustment. Alpha= 0,0083^.^ in case of PV+ cells. Kruskal-Wallis test was used to determine statistically significant differences.

|  | LI | LII/III | LIV | Lva | LVb | LVI |
| --- | --- | --- | --- | --- | --- | --- |
| LI |  | 0,0001 | 0,0001 | 0,0001 | 0,0001 | 0,0001 |
| LII/III |  |  | 0,361 | 0,0001 | 0,0001 | 0,0001 |
| LIV |  |  |  | 0,0001 | 0,0001 | 0,0001 |
| Lva |  |  |  |  | 0,008 | 0,311 |
| LVb |  |  |  |  |  | 0,0001 |
| LVI |  |  |  |  |  |  |

**Supplementary table 3:** This table shows the levels of significance (p) for a comparison of layers. Alpha was calculated by Bonferroni adjustment. Alpha= 0,0033^.^ in case of SST+ cells. Kruskal-Wallis test was used to determine statistically significant differences.

|  | Column vs. Septum |
| --- | --- |
| LI | 0,905 |
| LII/III | 0,825 |
| LIV | 0,0001 |
| Lva | 0,373 |
| LVb | 0,074 |
| LVI | 0,421 |

**Supplementary table 4:** This table shows the levels of significance (p) for a comparison of septal vs barrel compartments. Alpha was calculated by Bonferroni adjustment. Alpha= 0,0083^.^ in case of SST+ cells. Kruskal-Wallis test was used to determine statistically significant differences.

|  | LI | LII/III | LIV | Lva | LVb | LVI |
| --- | --- | --- | --- | --- | --- | --- |
| LI |  | 0,0001 | 0,0001 | 0,0001 | 0,106 | 0,569 |
| LII/III |  |  | 0,0001 | 0,0001 | 0,0001 | 0,0001 |
| LIV |  |  |  | 0,0001 | 0,0001 | 0,0001 |
| Lva |  |  |  |  | 0,0001 | 0,0001 |
| LVb |  |  |  |  |  | 0,001 |
| LVI |  |  |  |  |  |  |

**Supplementary table 5:** This table shows the levels of significance (p) for a comparison of layers. Alpha was calculated by Bonferroni adjustment. Alpha= 0,0033^.^ in case of VIP+ cells. Kruskal-Wallis test was used to determine statistically significant differences.

|  | Column vs. Septum |
| --- | --- |
| LI | 0,233 |
| LII/III | 0,049 |
| LIV | 0,0001 |
| Lva | 0,373 |
| LVb | 0,482 |
| LVI | 0,278 |

**Supplementary table 6:** This table shows the levels of significance (p) for a comparison of septal vs barrel compartments. Alpha was calculated by Bonferroni adjustment. Alpha= 0,0083^.^ in case of VIP+ cells. Kruskal-Wallis test was used to determine statistically significant differences.
