# Supplementary material for: Distribution Patterns of Three Molecularly Defined Classes of GABAergic Neurons Across Columnar Compartments in Mouse Barrel Cortex
Source: Front Neuroanat. 2019 Apr 30;13:45. doi: 10.3389/fnana.2019.00045 (PMC6503091; doi:10.3389/fnana.2019.00045)
Supplement: Supplementary file 2 [file Data_Sheet_1.PDF]

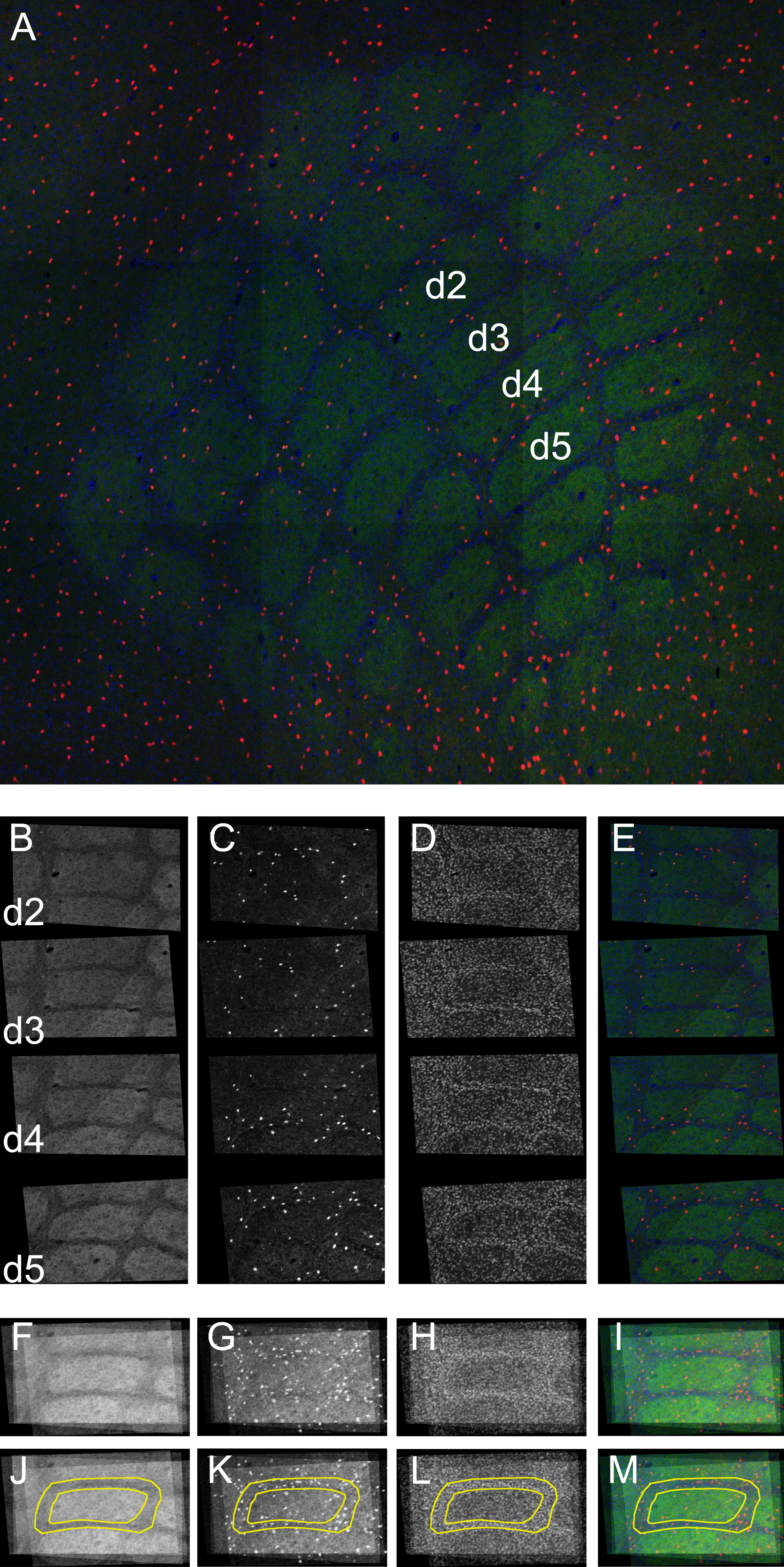

A
d2
d3
d4
d5
B
C
D
E
d2
d3
d4
d5
F
G
H
I
J
K
L
M

Supplementary Figure 1: (A) Montage of several images depicting the whole barrel field of a flattened cortex, which was cut in 50 micron thick sections parallel to the pial surface. The image shows a plane through layer IV, in which the barrels were located. It is important to note that the cell counting and delineation of the barrels were performed on this and comparable images. The projections as seen in Fig. 2 were also made of appropriate segments of such large images, but the projection images solely served a demonstration purposes, the cell counting was performed independent from them. B-D) Individual channels of selected (d2-d5) barrels, which were oriented and aligned similarly, to demonstrate the vGluT2 (B), SST (C) and DAPI (D) staining. E) Merged and pseudocolored images. The channels B and D clearly show the barrels, whereas the channel C displays the SST cells, which seemingly do not have any spatial preference. Projecting the single image planes into one plane (F-I) showed a similar barrel pattern (F, H, I), but it also showed the higher occurrence of SST cells in septal regions. The location preference of SST cells was even more obvious when the barrel and septal borders were projected on each of the images (J-M). In this case, only 4 barrels were use to demonstrate the working process, whereas in Figure 2, 15-20 such images were overlayed.

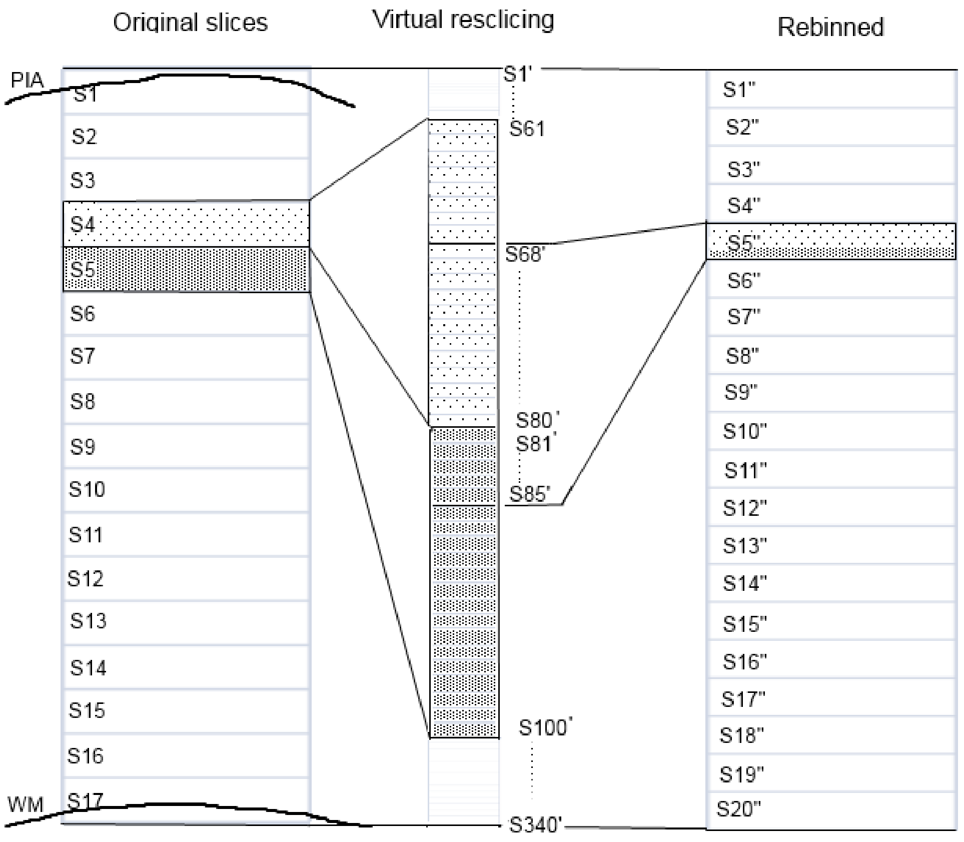

Supplementary Figure 2: Flow chart of the re-binning procedure for the normalization of tangential slices. In this example we originally had n=17 sections (S1-S17). Then, we virtually resliced the brain into n'= n* 20= 17* 20= 340 subsections (S1'-S340'). At the end, we created 20 normalized sections (S1"-s20").

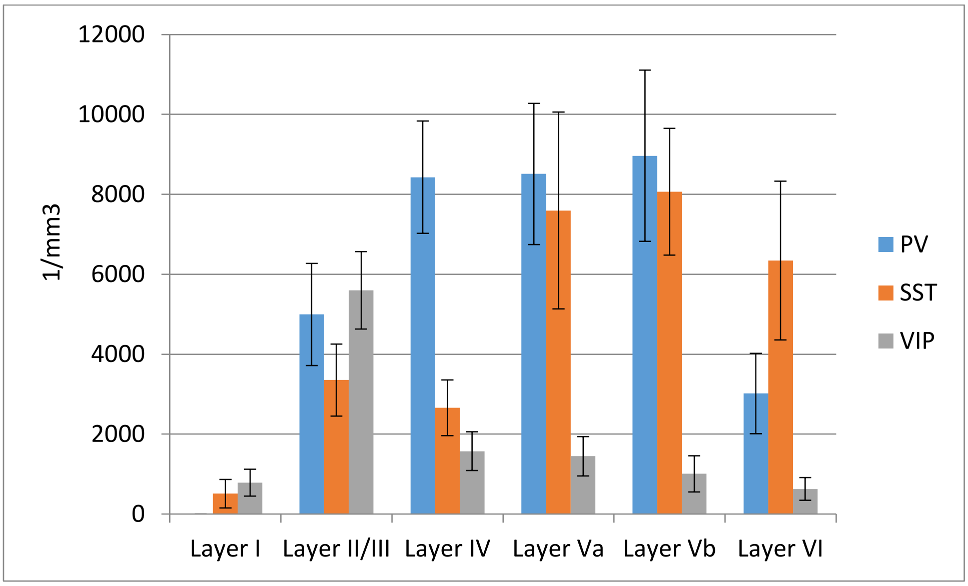

Supplementary Figure 2A: Densities in barrel columns: average cell density; whiskers: standard deviation

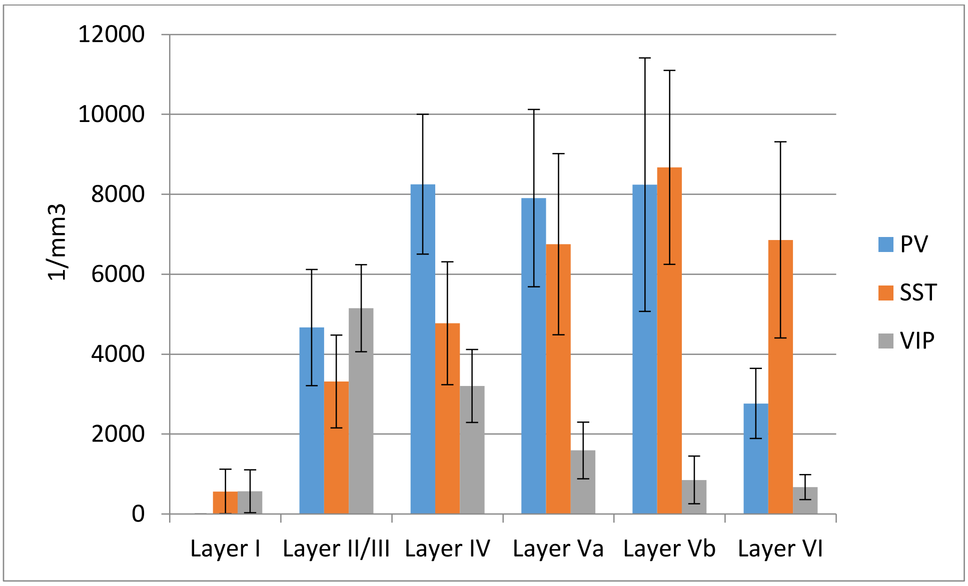

Supplementary Figure 2B: Densities in septa: average cell density; whiskers: standard deviation

	Columns	Septa
LI		
LII/LIII	0.0001	0.0001
LIV	0.0001	0.0001
Lva	0.037	0.005
LVb	0.039	0.979
LVI	0.002	0.0001

P-values of PV and SST density comparison

	Columns	Septa
LI		
LII/LIII	0.123	0.428
LIV	0.0001	0.0001
Lva	0.0001	0.0001
LVb	0.0001	0.0001
LVI	0.0001	0.0001

P-values of PV and VIP density comparison

	Columns	Septa
LI	0.021	0.966
LII/LIII	0.0001	0.0001
LIV	0.0001	0.0001
Lva	0.0001	0.0001
LVb	0.0001	0.0001
LVI	0.0001	0.0001

P-values of SST and VIP density comparison
